# Complementary Spiritist Therapy: Systematic Review of Scientific Evidence

**DOI:** 10.1155/2011/835945

**Published:** 2011-05-11

**Authors:** Giancarlo Lucchetti, Alessandra L. Granero Lucchetti, Rodrigo M. Bassi, Marlene Rossi Severino Nobre

**Affiliations:** ^1^Research Department, São Paulo Medical Spiritist Association, Avenida Juriti 367 SP Apto 131, 04520-000 São Paulo, SP, Brazil; ^2^Research Department, Brazilian and International Medical Spiritist Associations, Rua Pedro Severino, 323 - 1° Andar, 04310-060 São Paulo, SP, Brazil

## Abstract

Spiritism is the third most common religion in Brazil, and its therapies have been used by millions worldwide. These therapies are based on therapeutic resources including prayer, laying on of hands, fluidotherapy (magnetized water), charity/volunteering, spirit education/moral values, and disobsession (spirit release therapy). This paper presents a systematic review of the current literature on the relationship among health outcomes and 6 predictors: prayer, laying on of hands, magnetized/fluidic water, charity/volunteering, spirit education (virtuous life and positive affect), and spirit release therapy. All articles were analyzed according to inclusion/exclusion criteria, Newcastle-Ottawa and Jadad score. At present, there is moderate to strong evidence that volunteering and positive affect are linked to better health outcomes. Furthermore, laying on of hands, virtuous life, and praying for oneself also seem to be associated to positive findings. Nevertheless, there is a lack of studies on magnetized water and spirit release therapy. In summary, science is indirectly demonstrating that some of these therapies can be associated to better health outcomes and that other therapies have been overlooked or poorly investigated. Further studies in this field could contribute to the disciplines of Complementary and Alternative Medicine by investigating the relationship between body, mind, and soul/spirit.

## 1. Introduction

According to the latest Brazilian Demographic Census [1], “Spiritism” is the third largest religion in Brazil with approximately 2.3 million followers. Its believers make up the most well-educated (60% have at least 11 years of education) and wealthy populations in Brazil. 

Spiritism was founded by the French teacher and educator Hippolyte Léon Denizard Rivail also known by his pseudonym Allan Kardec [2]. From the outset, Rivail became interested in the wildly popular phenomenon of spirit tapping and decided to carry out his own research. He compiled a list of questions and began working with mediums and channelers to put them to spirits. 

In 1857, he published his first book on Spiritism entitled, “The Spirits' Book” [3], comprising a series of 1019 questions exploring matters concerning, for example, the nature of spirits. During his life, Rivail always strove to link experimental science with religion [4] and defined Spiritism as “a science which deals with the nature, origin and destiny of Spirits, as well as their relationship with the corporeal World” [5].

Since its emergence Kardec tried to use rigorous methods, such as those reported in Revue Spirite [6], “To this end, we gather facts, examine them, scrutinize them in painstaking detail, comment on them, discuss them objectively, without over-enthusiasm, and this was how we came to discover the admirable links among all elements of this vast science which address the most serious issues of humanity.”

Furthermore, Kardec stated [7], “Spiritism proceeds in the same way as the positive sciences, by using the experimental method. When facts of a new kind are observed, facts that cannot be explained by known laws, it observes, compares and analyzes them. Reasoning then from the effects to the causes, it discovers the laws which govern them.” 

Another example of the scientific aspect of Spiritism can be noted in “The Genesis” [8, 9], “science without Spiritism finds itself utterly powerless to explain certain phenomena by laws of matter alone; - while Spiritism without science would lack support and control.” 

Spiritism adopts a dualistic concept of the human being. It postulates that we are, essentially, immortal spirits that temporarily inhabit physical bodies for several necessary incarnations to attain moral and intellectual improvement. It also implies a possible beneficent or maleficent influence of the spirits over the incarnate human. In the case of maleficent influence, it is called “obsession” and it could be the cause of mental imbalance or cases of “madness” [10]. 

With the Spiritist Doctrine emerged the Complementary Spiritist Therapy [11], based on a range of therapeutic resources including prayer, laying on of hands, fluidotherapy (fluidic water or magnetized water), charity, spirit education, and disobsession (spirit release therapy). The name “Complementary” derives from the fact that Spiritist Therapy is used in conjunction with conventional medicine and not in place of it [12]. 

According to recent studies [13], there are more than 1650 Spiritist centers and numerous Spiritist hospitals throughout Brazil. Furthermore, practitioners of other religions (such as Catholics, Protestants, Jews, and others) make use of the Spiritist centers for spiritual treatment in order to cure or relieve their symptoms. In view of the numbers of people attending these centers, allied with the current expansion of their popularity, and the fact that some centers have been developing their protocols for more than 131 years, it is fair to assume that the protocols used have enjoyed considerable success. The Federation for Spiritism in Sao Paulo, Brazil serves between 7000 and 10000 people daily. Most of these people come to attend classes and/or receive spiritual healing. All Spiritist treatments are provided free of charge. 

There are also thousands of these centers in Spanish-speaking countries in Latin America and more than 161 centers in 31 countries outside Latin America, including more than 70 in the United States [13].

However, few studies, if any, have evaluated the efficacy of the healing practices of Spiritist centers in randomized double-blind controlled trials.

The aim of this paper was to examine the scientific basis for some Spiritist therapies in terms of health outcomes. This was a systematic review which focused on those studies that provided the most robust methodologies and thus presented the lowest likelihood of bias and confounding. Hypotheses supported by strong and consistent evidence have been highlighted, and gaps in the literature representing opportunities for future research were also identified. 

We identified six hypotheses that underlie most of the recommended Spiritist therapies, based on the Spiritist literature available. These hypotheses were as follows (1) prayer for oneself is associated with better health outcomes; (2) laying on of hands is associated with better health outcomes; (3) magnetized water is associated with better health outcomes; (4) volunteerism is associated to better health outcomes; (5) virtuous life and positive affect are associated with better health outcomes; (6) spirit release therapy is associated with better health outcomes.

## 2. Methods

### 2.1. Search Strategy

We carried out a search of published studies, without language restriction, using the Medline database (January, 1966, to March, 2010). Keywords used (Table 1) for each hypothesis were as follows: (1) prayer for oneself: “Prayer,” “Pray,” “Religious Meditation”; (2) laying on of hands: “Laying on of hands,” “Imposition of hands,” “Therapeutic touch,” “Reiki,” “Johrei”; (3) fluidotherapy: “fluidic water,” “Magnetized water,” “Water healing,” “Water heal,” “Energized water”; (4) charity: “Volunteering,” “Helping others”; (5) spirit education (Virtuous life and Positive affect): “Moral values,” “positive affect,” “anger proneness,” “Resilience” and “prospective,” “positive emotions,” “Emotional style,” “Goodness not Goodness-of-fit,” “Benevolence,” “Humility,” “Virtues,” “Peacefulness,” “Kindness”; (6) disobsession (spirit release therapy): “Possession Trance,” “Spirit Release therapy,” “Spirit possession,” “spiritist healers,” “spiritist healing,” “spiritual surgery,” “spiritist,” “spiritists.” We decided to limit the searches with the function [title] which retrieves only articles whose titles contain these specific words. 

Citations were also screened and full reports of potentially relevant studies obtained. We applied inclusion and exclusion criteria and assessed methodological criteria.

### 2.2. Study Exclusion Criteria

In line with a previous article based on systematic reviews on spirituality/religiosity [14], studies were categorized as inconclusive and subsequently eliminated from further consideration if their designs made it impossible to rule out bias, confounding, or chance as alternative explanations for results. Specific exclusions were made for any of the following criteria.

- *No attempt to control for any potential confounder*: studies that reported a univariate association with no attempt to control for such basic confounders as age, gender, or ethnicity were eliminated.
- *Cross-sectional design*: cross-sectional studies are unable to determine the temporal sequence of events.
- *No statistical analyses*: studies that failed to conduct statistical analyses did not assess the role of chance in accounting for the observed association.
- *Earlier reports on the same cohort*: series of reports are often conducted on a specific therapy-health link covering the same cohort differing only in terms of length of followup.
- *No evaluation of health outcomes*: some studies report only findings related to the opinions of health care professionals on a given issue.
- *Review or theoretical studies*: studies that proposed theories or hypothesis were excluded, as were narrative reviews.
- *Experimental studies*: explaining the mechanisms by which some therapies work.

### 2.3. Criteria for the Evaluation of Included Studies

Besides number of patients, studies were categorized as case-control or cohort, and if cohort, they were either prospective or retrospective. Prospective cohort studies were defined as those that identified a group of participants and followed them over time, whereas retrospective cohort studies were defined as those that identified a group of participants and used existing records to evaluate their clinical characteristics and course. The Newcastle-Ottawa scale [15] was used to assess the quality of each study (cutoff >6 adopted [16]). This measure assesses the methodology aspects of observational studies and encompasses the variables study quality, including selection of cases, comparability of populations, and ascertainment of exposure to risks. In all cases, disagreements among raters were resolved through discussion to reach a final consensus. 

Clinical trials were rated using the Jadad score [17]. This score is based on a scoring system to assess the methodological quality of clinical trials. Essentially it evaluates randomisation, blinding, and the mention of dropouts. Trials are scored on a scale of one to five, with higher scores implying higher quality. We decided to exclude all papers on the topic with a Jadad score of 3 or less.

### 2.4. Outcomes of Interest and Definitions

Health outcome was defined in the present study as “a change (or lack of change) in health status caused by a therapy or factor compared with a previously documented health status using disease-specific measures, general quality of life measures or utility measures.”

The following outcomes were used to compare the users and nonusers of some principles of Spiritist therapy, based on the literature available after article selection.

- Prayer for oneself: hospitalization, complications after coronary artery bypass graft surgery (CABG), quality of life, stress, depressive symptoms, optimism, and general distress.
- Laying on of hands: behavioral symptoms, fatigue, pain, anxiety, satisfaction with life, and functional status.
- Fluidotherapy (magnetized water): dental calculus (this was the only health outcome evaluated by good methodological studies).
- Charity/volunteering: physical activity, self-reported health, depression, mortality, well-being, functional disability and life satisfaction.
- Positive affect and virtuous life: cardiovascular outcomes (peripheral artery disease, coronary heart disease), mortality, frailty, self-reported health status, functional status, remission from personality disorders, distress, HbA1c levels, well-being, satisfaction with life, happiness, pain, anger and psychological distress, and social connection.
- Disobsession (spirit release therapy): no studies were included in the final analysis regarding this issue.

### 2.5. Data Abstraction

**Data Abstraction Consisted of 3 Phases**

**Phase 1**

Two researchers (GL, ALGL) independently screened the list of references (full articles were retrieved for further analysis whenever necessary) to exclude reports not assessing the issue in-hand, not assessing the relationship between different types of therapies and health outcomes, as well as articles not containing abstracts.

**Phase 2**

Each study was characterized according to: year of publication; country of origin and population evaluated; length of followup; number, agem and gender of the participants, and health outcome. All articles not fulfilling the inclusion criteria and which met the exclusion criteria were omitted from the final analysis.

**Phase 3**

Another extensive revision of inclusion and exclusion criteria were performed, and the quality of each study was assessed by the Jadad score for clinical trials and the Newcastle-Ottawa scale for cohort studies. Two researchers (GL and ALGL) rated the articles. The disagreements between the reviewers were discussed with a third reviewer (RMB) and resolved by consensus.

### 2.6. Statistical Analysis

This paper uses the term systematic review to denote the entire process of retrieving, selecting, appraising, summarizing and reporting evidence, and performing the meta-analysis for the specific statistical technique of combining the data from individual studies.

Due to the nature of this systematic review (the studies are dissimilar with respect to population, outcome, and intervention), no meta-analysis could be performed. Instead, we opted to present those articles supporting each intervention or otherwise, along with the quality of these studies.

## 3. Results

We identified 1998 articles in the preliminary analysis (Table 1). Following the first phase, 1752 were excluded for not assessing the topic analyzed or investigating the association between the therapy and health outcome. Of the 246 articles remaining in Phase 2, 138 were excluded (most studies did not evaluate outcomes, had sample problems, or dealt with intercessory prayer), giving 109 articles.

Finally, further 59 articles were excluded in Phase 3, giving a total of 45 for the final analysis (6 for prayer, 11 for laying on of hands, 2 for fluidic/magnetized water, 10 for charity/volunteering, 21 for spirit education (virtuous life and positive affect), and 0 for disobsession). 

Out of the 11 articles regarding prayer for oneself in phase 3, only 6 were included for final analysis (with the exclusion of 2 articles with Newcastle-Otawa scale <7; 1 cross-sectional; 1 recorded prayer and 1 experimental study).

Of the 35 studies regarding laying on of hands in Phase 3, 11 were included for final analysis (with the exclusion of 6 noncontrolled trials, 11 with nonmimic therapeutic touch control, and 7 experimental studies). The 2 studies on fluidic/magnetized water in phase 3 were included. 

Of the 12 studies on volunteering in Phase 3, 10 were included for final analysis (2 cross-sectional studies were excluded in this phase), and of the 37 studies assessing spirit education (virtuous life and positive affect), 21 were included (with the exclusion of 7 with Newcastle-Ottawa <7; 2 not evaluating health outcomes; 7 experimental; 5 positive affect as mediator, not predictor) and 2 with Jadad score less than 4 (from loving-kindness meditation).

The scientific basis for each Spiritist therapy affecting different health outcomes is outlined below.

## 4. Prayer for Oneself

**Hypothesis 1**

Prayer for oneself is associated with better health outcomes.

**Background**

The first resource is to pray. The National Center for Complementary and Alternative Medicine in the United States has defined prayer as an active process of communicating with and appealing to a higher spiritual power. According to Amellin [18], 82% of Americans believe that prayer can cure serious illness, 73% believe that praying for others can cure illness, and 64% want their physicians to pray with them. Along the same lines, McCraffey et al. [19] showed that 69% of those praying for specific medical conditions found prayer very helpful.

**Spiritist View**

According to Spiritism [20, 21], “It is through prayer that Man obtains the assistance of the good Spirits who come running to sustain him in his good resolutions and inspire wholesome ideas …. By these means he can also turn away from himself all the evil which he attracts through his faults.” Furthermore, “Through prayer, the donor casts out from their own midst, the shadows which gather in their daily striving, whilst absorbing renovating substances for their own well-being from the spiritual plane” [22, 23].

**Scientific Evidence**

Six studies scored more than 6 points on the Newcastle-Ottawa scale and were included in the final model (Table 2). All studies evaluated prayer for oneself. We decided not to include some studies on intercessory prayer given its major and intractable methodological flaw, namely, that receipt of prayer cannot be controlled and therefore it is impossible to ascertain to what degree individuals in the control groups were actually the recipients of the “intervention” from loved ones, family members, clergy, or others, besides the research intercessors. For further reading, consult the recommended literature [24].

Examining the studies selected, Ai et al. [25] found that higher prayer frequencies were associated to reduced complications following coronary artery bypass graft surgery but not to length of hospital stay. This finding seems especially significant considering that a behavioral aspect could be exerting an influence on an important physical outcome.

The same group of researchers evaluated patients post-CABG with promising results. In 2002 [26], the group evaluated prayer for oneself and found that it was directly associated with optimism and healthier effect. The same was noted in 2007 when prayer coping was correlated to less preoperative stress symptoms [27]. Notably, even after a one-year followup, results of prayer were maintained including lower rates of depression and general distress [28].

A study examining the relationship between prayer and quality of life pointed to a mediation of cognitive coping and perceived social support [29].

Nevertheless, another study conducted in 2007 [30] evaluating community elderly found that prayer frequencies did not predict change in depressive symptoms after 3 years of followup.

**Study Limitations**

There are several limitations with respect to prayer studies. First, prayer can have an adaptative and/or a maladaptative coping response. For example, praying with an attitude of waiting for a miracle to happen or relying on doctors to find a cure for one's ailment can have a negative impact on health whereas active religiousness can have positive outcomes. Thus, it is necessary to evaluate whether prayer in fact has a negative or positive impact on the person. Second, prayer is not standardized, and approaches can vary depending on the religious affiliation. Third, some measures of prayer, such as prevalence of prayer and frequency of prayer, are simplistic measures which may not reflect the whole process of faith and coping.

**Future Directions**

In our systematic review, concerns over the lack of studies should be highlighted. The majority of cases were cardiological patients while only one study evaluated community elderly [30]. We believe that the numerous studies in the literature dealing with intercessory prayer should be superseded instead by evaluating praying for oneself (easy to control and represents active religiousness). The long-term benefits or malefits of such “intervention” should be analyzed through representative population cohort studies designed with this specific objective, and not as a subpart of larger studies.

**Conclusion**

Prayer for oneself appears to be linked to better health outcomes such as lower rate of complications after CABG, improved optimism, and reduced stress. However, lack of rigorous methodological studies and highly selected populations preclude confirmation of this hypothesis at present.

## 5. Laying on of Hands

**Hypothesis 2**

Laying on of hands is associated with better health outcomes.

**Background**

Also known as an imposition of hands or laying on of hands, the therapeutic touch is a common issue in medicine and nursing today. Some authors have defined this under “biofield therapies” which “are those modalities including Reiki, *qigong* therapy, and Therapeutic Touch, among others, performed by trained practitioners who manually and/or via intent, interact with energy fields of the patient” [31].

Biofield therapies are among the most widely used complementary therapies, and demand for them has been growing [32]. The interest in this issue prompted the University of Arizona to create the “Center for Frontier Medicine in Biofield Science.”

**Spiritist View**

According to Spiritism, laying on of hands is defined as “an energy transfusion, changing the cellular field” [22, 23] and “a psychic energy transfusion” [33, 34]. Further, regarding imposition of hands energies [35, 36], “The spiritual fluids, which constitute one of the states of the universal cosmic fluid, are then the atmosphere of spiritual beings. It is the element whence they draw the materials with which they operate,—the place where special phenomena take place, perceptible to the sight and hearing of the spirit, but which escapes the carnal senses which are impressed alone by tangible matter.” Kardec also pointed [7], “Fluidic matter produces a similar effect to medicinal matter, only with a difference (owing to the former's greater depth of penetration given the tenuity of its constituents) of acting more directly on primary molecules of the organism than on larger molecules of tangible matter …. Its qualities are affected by thought.”

**Scientific Evidence**

In the following analysis, we decided to include only comparative studies evaluating therapeutic touch (TT)/laying on of hands (LOOH) and sham (mimic) TT/LOOH. Groups not submitted to TT/LOOH or mimic TT/LOOH were excluded due to the placebo effect of imposition of hands. A total of 11 clinical trials were retrieved for the final analysis (Table 3).

Three studies were conducted in long-term care and nursing home residents, showing reduced behavioral symptoms in dementia patients including restlessness, stress (lower morning cortisol) [37], physical nonaggressive behaviors [38], and wandering, tapping, banging, pacing, and walking [39].

Another seven studies evaluated pain measures. Results indicated decreased pain in patients with cancer undergoing chemotherapy [40] as well as those with knee osteoarthritis [41], burns [42], and headache [43]. Nevertheless, some studies revealed a nonsignificant difference in patients submitted to stereotactic biopsy [44], carpal tunnel [45] and those experiencing postoperative pain [46].

Regarding anxiety, two studies showed no difference between TT and mimic TT [44, 47], and one study observed improvement on the visual analogic scale for anxiety in burn patients [42].

Additionally, in order to minimize the placebo effect, *in vitro* studies were performed showing an increase in human cells in cultures [48], DNA synthesis and mineralization of human osteoblasts [49], and elevated blood hemoglobin and hematocrit Levels [50].

**Study Limitations**

Therapeutic touch studies have been widely reported in the medical literature. However, most studies involved small, convenient samples and had nonrandomized controlled and blind designs. Another concern is the short followup (days), evaluating only acute outcomes such as pain and anxiety. Moreover, many studies used visual analogic scales preventing more complex analysis of this field.

**Future Directions**

In our opinion, future studies should focus on the differences among therapeutic touch, a defined technique developed by Krieger [51], and other types of hand imposition such as the Spiritist “passe,” Johrei and Reiki. Another aspect that should be evaluated is the comparison of intentional laying on of hands and healing techniques based on a “high healing” power. For example, a study conducted in Austria [52] sought to assess the difference in efficacy between a healer and an actor. However, the healer became unblinded and withdrew from the study after the first session, and therefore the results are misleading. This was a valuable attempt to understand the role of therapies including Johrei, Heike, and Spiritist “passé.” Furthermore, another future direction is focusing on the identification of which types of energies were irradiated from the imposition of hands. The chronic use of TT should also be evaluated in representative populations.

**Conclusion**

The body of evidence accumulated to date points to a positive effect of therapeutic touch on behavioral symptoms and in most pain studies, while presenting contradictory results regarding anxiety. Some experimental studies have also shown promising results on cells (human cultures, osteoblasts, red blood cells) further supporting this hypothesis.

## 6. Fluidotherapy (Fluidic Water)

**Hypothesis 3**

Magnetized water is associated with better health outcomes.

**Background**

*The concept of magnetized water was studied by Masaru Emoto, whose team carried out a study *which was tested under double-blind conditions. The theory that water “treated” with an intention can affect ice crystals formed from that water was tested, and the results revealed that crystals produced from the treated water were given higher scores for aesthetic appeal than those from the control water (*P* = .001) thus lending support to the hypothesis [53]. In 2004, the same author [54] wrote an article entitled, “Healing with water” published in the Journal of Alternative and Complementary Medicine, describing the power of healing through water.

**Spiritist View**

According to the Spiritist view [35, 36], “the most simple agents—water, for example—can acquire powerful and effective qualities under the action of the spiritual or magnetic fluid, for which they serve as vehicle, or reservoir.” Furthermore, Spirit Emmanuel in the book “Follow me” (“Segue-me”) [55] stated, “- Water can acquire subtle or “fluidic” qualities upon being induced by the agent of Will. Magnetized or fluidified water. The agent of fluidified water consists of the properties instilled in it and possesses medicinal power which acts on the perispirit and indirectly contributes to restoring the physical body.”

**Scientific Evidence**

Only two studies remained after applying of rigorous exclusion criteria. Curiously, both were from the odontology field. In 1998, Johson et al. [56] evaluated the effects of a magnetized water oral irrigator on plaque, calculus, and gingival health. The result showed that the group using magnetized water resulted in 64% less calculus in comparison to the control group where this reduction reached statistical significance, corroborating the results found by Watt et al. [57].

Some experimental studies were conducted on this issue. In Brazil, a study carried out by Savieto et al. [58] evaluated the effects of the consumption of water treated with therapeutic touch on the healing of uniform wounds in the skin of mice. The average wound size in the experimental group was found to be consistently smaller than that in the control group, and the difference among the measures obtained in the two groups reached statistical significance. The authors' conclusion was that the water undergoing therapeutic touch can serve as a complementary therapy in wound treatment. Similar results were found in other two studies from the same authors [59, 60]. In China, a study conducted by Yi-Long et al. [61] studied the effect of magnetized water on enzyme activities and showed that the activity of glutamate decarboxylase was increased by 30% in magnetized water.

However, few studies have assessed this hypothesis in humans. None of the articles on studies in humans were selected in this systematic review due to serious methodological issues. Nevertheless, we decided to cite some references. Merkulova and Mikhel'son [62] from Russia tested the action of magnetized water on the quality of health at a sanatorium, reporting positive results, and also tested the biological and bactericidal action of magnetized water [63]. In 1987, scientists from China tested the effect of magnetic water on urinary calculi [64] and for prevention of diarrhea [65] reporting successful outcomes.

**Study Limitations**

Several limitations should be outlined pertaining to the studies focusing on this hypothesis. First, there is a lack of sound methodological studies in the field. Second, few researchers are currently studying this issue, and third, there are a huge number of pseudoscientific statements made to the lay public, hampering a more serious analysis.

**Future Directions**

There are few studies in the literature assessing this issue. Most studies are based on noncontrolled observational studies conducted between 20 and 30 years ago. It seems that fluidic (magnetized) water has been left to the wayside in modern medical science. Such a simple procedure must be analyzed with serious scientific rigor in order to confirm beneficial effects or otherwise. The future direction for this field should be based on well-conducted clinical trials in laboratory animals and humans. There are no contraindications for the use of water, and in addition it represents a low-cost and highly accessible resource.

**Conclusion**

There is a lack of well-conducted controlled, double-blind studies on magnetized/fluidic water. Some observational data from the 1980s points to potential beneficial effects in humans, and studies in rats have shown promising results. However, currently available evidence neither supports or rejects this hypothesis. Clearly, further studies are warranted on this issue.

## 7. Charity (Volunteerism)

**Hypothesis 4**

Volunteerism is associated to better health outcomes.

**Background**

The association between charity and health is gaining increasing scientific recognition. Charity is defined by the Merriam-Webster dictionary as, “benevolent goodwill toward” and “generosity and helpfulness especially toward the needy or suffering.” Post [66] wrote an article called “It's Good to be Good: Health and the Generous Heart” where he believes that “the evidence supports that one of the healthiest things a person can do is to step back from self-preoccupation and self-worry, as well as from hostile and bitter emotions, and there is no more obvious way of doing this than focusing attention on helping others.”

In the early 1970s, the “helper therapy” principle was noted in a few leading psychiatry journals as professional researchers found that helping others was beneficial in a variety of settings. Luks [67], in a survey of thousands of volunteers across the United States, found that people who helped others consistently reported better health than peers in the same age group.

**Spiritist View**

Kardec wrote [68], “without charity, there is no salvation” and that the “greatest model of charity and moral perfection that we have to follow is Jesus” [3]. Charity according to Spiritism [3] is, “Benevolence for everyone, indulgence for the imperfections of others, forgiveness of injuries.”

**Scientific Evidence**

Ten studies scored more than 6 points on the Newcastle-Ottawa scale and were included in the final model (Table 4). All studies were cohort type allowing cause-effect conclusions. Three studies analyzed the relationship between volunteering and mortality. Those who frequently volunteered had significantly reduced mortality compared to nonvolunteers [69, 70]. Additionally, years of volunteering were also associated with lower mortality. In a study carried out on a 7-year cohort, those who volunteered for 10 to 14 years had a lower mortality risk compared to nonvolunteers [71]. These results remained statistically significant even after controlling for sociodemographic aspects such as, age, sex, income, race and also for physical and social aspects.

Most studies point to an association between volunteering and better self-reported health [72–75], well-being [74], and life satisfaction [76]. This relationship includes a 47-year followup of 4000 women and men graduated from Wisconsin high schools [74]. According to the authors, “Using the Wisconsin Longitudinal Study, we have once again demonstrated that volunteering is positively related to psychological wellbeing. In addition, we provide strong evidence that the relationship is *causal*, based on analyses controlling for well-being in 1992 when predicting essentially the same measure in 2004.”

Some articles analyzed the association between mood disorders, especially depression. Two studies [72, 75] show an inverse relationship between volunteerism and depression in the adult population, independently of age while two studies [77, 78] demonstrated the same association in elderly. Particularly in depression, older persons seem to benefit more from volunteering as an alternative to retirement.

Finally, two studies were conducted regarding physical health. Volunteers tend to have less functional disability [75] and engage more in physical activity [72].

**Study Limitations**

Some self-reported health measures such as self-rated health and well-being may not adequately analyze the complexities of the human state. We believe that the use of validated and multidimensional scales could enable better representation of these aspects. Another limitation is that volunteerism includes a variety of different aspects, such as church-based, financial, and social volunteerism, that should be analyzed separately and not together as a whole. Furthermore, the majority of studies, not reported here, are cross-sectional and therefore do not examine cause-effect relationships.

**Future Directions**

A future direction for this field is the analysis of which mechanisms are involved in this causal relationship. Is it the good feeling that decreases cortisol or proinflammatory levels and reduces mortality in this sample? Or could another mechanism be involved? This represents a vast area for future research. Another future line of research entails analyzing other factors such as stress, anxiety, phobic disorders, hospitalization, and cerebro-cardiovascular outcomes.

**Conclusion**

Evidence supporting a relationship between volunteerism and health outcomes is moderate to strong, particularly concerning survival, well-being, health status and depression in elderly. The mechanisms underlying this relationship warrant further investigation.

## 8. Spirit Education (The Role of Virtuous Life and Positive Affect)

**Hypothesis 5**

Virtuous life and positive affect are associated with better health outcomes (Table 5).

**Background**

This field is gaining attention in the medical literature. A study by Williams et al. [79] showed that proneness to anger places normotensive middle-aged menand women at significant risk for CHD morbidity and death, independent of the established biological risk factors.

In his book “Forgive For Good,” Luskin [80] of Stanford University began studying Protestant and Catholic mothers whose children were killed in political-religious clashes. Forgiveness interventions lowered blood pressure, reduced stress hormones, improved depression, and enhanced self-reported health.

Similarly, Dillon and Wink [81] in their book “In the Course of a Lifetime” analyzed 300 preteens in the Bay Area followed every ten years since the 1920s. Those who identified compassion and generosity as important were healthier and happier 50 years later (protected from depression and some physical illnesses).

These moral values are responsible for a positive affect on dealing with life and its adversities.

**Spiritist View**

According to Spiritism, “the main purpose of a Spirit to return to a body of a child is to be educated again. Hence, the importance of the Spiritist Education, because to spiritually educate the children is to prepare them to face all times and all adversities of life according to the postulates of the Gospel” [82]. Jesus's lessons [68] include the cultivation of love, humility, purity, peace, goodness, mercy, benevolence, compassion, good feelings, among others. Kardec once wrote [3], “The cure may be slow, for the causes of the malady are many, but it is not impossible. It can only be effected, however, by going to the root of the evil, that is to say, by generalizing education; not the education which merely advances men in knowledge, but that which improves them morally. Education, rightly understood, is the key of moral progress, when the art of training the moral nature shall be understood as is the art of training the intellect, it will be possible to straighten a crooked nature as we straighten a crooked sapling.” Spiritism also believes in the power of positive thinking as stated in the following sentence: “Bad thoughts corrupt the spiritual fluids, as deleterious miasmas corrupt the air we breath [8, 9].”

**Scientific Evidence**

Twenty-one articles were selected for final analysis. Spirit education is based on moral values and seems to have a positive affect on dealing with life and its difficulties. There is strong support in the medical literature concerning positive affect on a range of health outcomes. Four studies from this systematic review analyzed the role of positive affect and mortality. All studies found a positive relationship between positive affect and overall survival in a representative sample of adults [83], diabetic patients [84], HIV patients [85], and Catholic nuns [86]. Only the comparison group of the study carried out in diabetic patients [84] found no association between positive affect and mortality. However, when the analysis was limited to those over the age of 65, specific positive affects, in particular hopeful and enjoyed life, were significantly associated with lower risk of mortality.

Furthermore, positive affect was related to fewer cardiovascular events in healthy adults [87] and in patients following percutaneous coronary intervention (PCI) [88] and to lower risk for frailty [89, 90], lower incidence of compromised activities of daily living in the elderly [91], improved performance-based functioning after hip fracture [92], better patient-reported health status post-PCI [93], and higher remission for personality disorders in adulthood [94].

Anger has been evaluated by two studies, finding anger proneness to be positively associated with incident peripheral artery disease [95], while trait anger was related to increased risk for coronary heart disease [79].

Resilience was related to lower distress and better HbA1C in diabetics [96] and predicted lower mortality in Israeli Jews [97].

A number of experimental studies support this hypothesis. In 2006 [98], volunteers were exposed to a rhinovirus or influenza virus by nasal drops and monitored for symptoms. Positive emotional style was associated with lower risk of developing an upper respiratory illness. Another study [99] reported that positive affect was inversely associated with cardiovascular reactivity, norepinephrine level, and morning rise in salivary cortisol in 328 healthy individuals during a sadness and an anger recall task.

Some studies also evaluated the role of virtuous life on health outcomes. Kindness was associated to better life satisfaction [100] and happiness [101], and a loving-kindness intervention was associated to a higher social connectedness [102] and lower back pain, anger and psychological distress [103]. Benevolence was also evaluated on a longitudinal study showing a positive relation with well-being [104].

**Study Limitations**

Most studies were based on the positive affect subscale of the CES-D (Center for Epidemiologic Studies Depression Scale). Other types of measurements should be employed in a bid to replicate these findings.

**Future Directions**

Most studies were based on positive affect or positive emotions. However, few studies assessed aspects that could foster positive affect such as benevolence, goodness, humility, peace, and compassion. Investigating the role of these moral values on health outcomes may change the view of health-disease mechanisms in the future. Additionally, better understanding the mechanisms underlying this relationship could elucidate some aspects of psychosomatic medicine.

**Conclusion**

There is strong support for the hypothesis that positive affect is associated with better health outcomes, especially survival, as well as lower risk of physical health disability and fewer cardiovascular events. Several experimental studies also corroborate this finding.

## 9. Disobsession (Spirit Release Therapy)

**Hypothesis 6**

Spirit release therapy is associated with better health outcomes.

**Background**

Although spirit possession cases have been reported in the medical literature [105–107], few articles on disobsession therapy have been published to date. According to a publication from the Royal College of Psychiatrists [108], “Release may be effected by encouraging the spirit to go back into the memory of its death and to understand the nature of its entrapment. This understanding, offered with empathy, usually leads to a letting go and the awakening of a desire to enter the light. This may happen on its own or accompanied by a loving spirit or group of spirits who approach and greet the lost spirit with that purpose.” The Diagnostic and Statistical Manual of Mental Disorders—Fourth Edition [109] states, “Possession trance, a single or episodic alteration in the state of consciousness characterized by the replacement of customary sense of personal identity by a new identity is attributed to the influence of a spirit, power, deity, or other person.”

According to the ICD-10 [110] (Classification of Mental and Behavioural Disorders): F44.3: trance and possession disorders mean disorders in which there is a temporary loss of both the sense of personal identity and full awareness of the surroundings; in some instances the individual acts as if taken over by another personality, spirit, deity, or “force.” Attention and awareness may be limited to or concentrated upon only one or two aspects of the immediate environment, and there is often a limited but repeated set of movements, postures, and utterances. Only trance disorders that are involuntary or unwanted and that intrude into ordinary activities by occurring outside (or being a prolongation of) religious or other culturally accepted situations should be included here.

**Spiritist View**

The Spiritist etiologic model for mental disorders includes the negative influences of discarnated spirits (termed “obsession”). In addition to conventional medical and psychological therapeutics, Spiritist séances for disobsession are recommended, as well as “passes” (laying on of hands), prayers, and efforts to live according to ethical principles [111]. In the “Gospel according to Spiritism [68],” “obsession is the persistent action which an inferior or bad Spirit exercises over an individual. It may present many varied characteristics, from a simple moral influence with no perceptible exterior signs, to a complete organic and mental perturbation …. In the same manner that sicknesses are the result of our physical imperfections, which make the body accessible to pernicious exterior influences, obsession is always the result of moral imperfections, which allow the access of a bad Spirit …. It is very important to free the person from these negative vaporous fluids.” Furthermore, in the book “Disobsession” [112], “*We reiterate that Spiritism is Restored Christianity and that the leading Pioneer in disobsession, through enlightening unhappy spirits and curing the afflicted of all ailments was indeed Jesus himself.*”

**Scientific Evidence**

Despite the number of articles on spirit possession, few studies have evaluated disobsession (spirit release therapy) and its relationship with health outcomes. Consequently, no studies were selected for our final analysis.

However, one study compared reported expectations and outcomes of mental health center patients and patients of Spiritist healers [113]. According to the author, the Spiritists' patients reported significantly higher expectations, especially for complaints regarding mood and feelings. Both patient groups had a similar duration and severity of symptoms. The outcome ratings of Spiritists' patients were significantly better than those of therapists', but this difference could be accounted for by the higher expectations of the Spiritists' patients. Nevertheless, this study had several limitations: noncontrolled and nonrandomized research, inadequate statistical analysis, comparison between conventional treatment versus alternative treatment yet not for the two associated. Unfortunately, no conclusions could be drawn from this study.

Another study [114], not indexed on the Medline database and thus not included in our final analysis, was conducted in Brazil. The authors evaluated 40 patients with mental disabilities (20 submitted to disobsession sessions and 20 controls, with usual care in both). The Interactive Observation Scale for Psychiatric Inpatients (IOSPI) was employed to evaluate outcomes. The comparison of the control group with the experimental group verified a difference in variation between the groups (*P* = .045), demonstrating possible benefits of this intervention.

Some studies have assessed psychiatric disorders in mediums. An interesting paper [115] studied the similarities and differences between Brazilian Spiritist mediums and North American dissociative identity disorder (DID) patients and found that, compared with DID patients, the mediums differed in having better social adjustment, lower prevalence of mental disorders, lower use of mental health services, no use of antipsychotics, and lower prevalence of histories of physical or sexual childhood abuse, sleepwalking, secondary features of DID, and symptoms of borderline personality. According to the authors, mediumship differed from DID in having better mental health and social adjustment, and a different clinical profile. Similarly, Kua et al. [116] analyzed 36 young men with possession-trance. At followup four to five years later, none of the 26 who could be contacted showed any evidence of mental illness.

Another study [117] evaluated 115 mediums selected from different Kardecist Spiritist centers in São Paulo, Brazil and found that mediums included in this study had a high socioeducational level, a low prevalence of mental disorders and were socially well adjusted. The mediumistic process was characterized by dissociative and psychotic experiences that were not related to mental disorders.

**Study Limitations**

Only one study assessed disobsession (spirit release therapy), based on a randomized, controlled and blinded trial. However, several methodological issues need to be addressed such as challenges in standardizing treatment, the many different religions that use these techniques, and the ideal frequency and duration of these sessions. Additionally, mediumship must be reliable (raising the questions: what is reliable mediumship scientifically and how can this be measured?), where the medium should be evaluated for psychiatric conditions such as schizophrenia.

**Future Directions**

We should highlight that these therapies are complementary and not substitutes for usual care. Thus, an intervention group can receive spirit release therapy plus usual care while the control group receives only usual care. Furthermore, many outcomes could be tested such as mental disorders, mortality, hospitalization, well being, and self-reported health.

Gaining a deeper understanding of spirit possession, how it works and how it can help patients, is necessary to further assess this hypothesis. At present, numerous questions remain unanswered in this field.

**Conclusion**

There is a lack of well-conducted controlled, double-blind studies concerning disobsession (spirit release therapy), precluding support or rejection of this hypothesis at the present time. Further studies are now needed in this field.

## 10. Discussion

This systematic review has some limitations that should be pointed out. First, it comprised an extensive review of a highly complex field, and thus some important keywords may have been omitted in the present analysis. Second, in spite of being the most known and one of the most important medical databases in the world, Medline may not hold all relevant studies in this field, leading to the omission of some studies in this paper. Third, the Newcastle-Ottawa and Jadad score are very reliable measures but have some limitations resulting in some sound studies being excluded.

We should also point up that most studies evaluated were observational and not RCT studies and these studies were not carried out in Brazil. Therefore, generalizability is limited, and replication is needed.

However, this paper indicates a new field of research not yet fully explored by medical science. It shows that science is indirectly demonstrating that some of these therapies are related with better health outcomes while other treatments have been overlooked or poorly assessed. Fresh studies in this field could help the discipline of Complementary and Alternative Medicine to investigate the relationship between body, mind, and soul/spirit. Furthermore, discussing and assessing new methodological approaches to conduct these studies are important in order to scientifically prove these associations or otherwise. 

Spiritism is present in more than 31 countries outside Latin America (including Porto Rico, United States, Portugal), and these therapies are based on simple and noncostly procedures. According to Bragdon [13] in her article “Spiritist healing centers in Brazil,” “There are millions of Spiritists who go about the work of *reforma intima*, channeling, healing, praying, and doing charity work—without commercialization, self-serving promotion, or flamboyant display. Their way of life may in fact be the essence of a healing life of the spirit: it is a way of life that allows the individual to become whole, more healthy, more knowledgeable, more giving, more connected to community and higher power, and consistently progressing on the path of spiritual evolution.”

According to Spiritism [118], “For Earthly man, health may mean perfect balance of material organs; on the spritual plane however, *health is the perfect harmony of the soul*, which often can only be achieved by the invaluable contribution of the transient ills and deficiencies of Earth.”

Although these therapies have been little studied and are poorly understood by modern day science, some initiatives such as the NUPES (Núcleo de Pesquisas em Espiritualidade e Saúde) by the Federal University of Juiz de Fora, Brazil, São Paulo, Brazilian and International Medical Spiritist Association, Brazil, PROSER (Programa de Saúde e Espiritualidade) from the University of São Paulo, Brazil and, João Evangelista Hospital, Brazil are trying to encourage further scientific research into spiritual healing protocols and to promote international congresses. 

Moreira-Almeida and Neto [111] wrote, “The importance of Spiritist views in Brazil indicates the need for more academic research on this tradition.” The present authors share this view. Now is the time to undertake a thorough investigation on spiritual treatments, regardless of religious affiliation or scientific dogmas. 

“Unshakable *faith* is only that which can meet reason face to face in every human epoch”—Kardec.

## Figures and Tables

**Table 1:** Data abstraction.

Keywords used (only articles with these words in the title were included)	Articles found	Excluded in Phase 1	Phase 2	Excluded in Phase 2	Phase 3	Excluded in Phase 3	Articles included for the final analysis
Prayer							
Prayer	428	384	44	33	11	5	6
Pray	39	38	1	1	0	0	0
Religious Meditation	0	0	0	0	0	0	0

Laying on of hands							
Laying on of hands	18	17	1	0	1	1	0
Imposition of hands	0	0	0	0	0	0	0
Therapeutic touch	261	220	41	12	29	18	11
Reiki	0	0	0	0	0	0	0
Johrei	9	1	8	3	5	5	0

Fluidotherapy							
Fluidic water	0	0	0	0	0	0	0
Magnetized water	7	1	6	5	1	0	1
Magnetic water	9	7	2	1	1	0	1
Water Healing	0	0	0	0	0	0	0
Water Heal	0	0	0	0	0	0	0
Energized Water	0	0	0	0	0	0	0

Charity							
Volunteering	242	216	26	14	12	2	10
Helping others	41	40	1	1	0	0	0

Spirit education							
Moral values	59	57	2	2	0	0	0
Anger proneness	5	3	2	0	2	0	2
Resilience and prospective	97	79	18	13	5	2	3
Positive Emotions	53	41	12	5	7	5	2
Positive affect	142	110	32	12	20	11	9
Emotional style	4	1	3	0	3	3	0
Goodness NOT, Goodness-of-fit	82	82	0	0	0	0	0
Benevolence	49	48	1	0	1	0	1
Humility	129	128	1	1	0	0	0
Virtues	215	214	1	1	0	0	0
Peacefulness	1	1	0	0	0	0	0
Kindness	125	119	6	0	6	2	4

Disobsession							
Possession Trance	3	1	20	19	1	1	0
Spirit Release Therapy	0	0	0	0	0	0	0
Spirit Possession	34	28	6	5	1	1	0
Spiritist Healers	0	0	0	0	0	0	0
Spiritist Healing	2	0	2	1	1	1	0
Spiritual surgery	0	0	0	0	0	0	0
Spiritist	8	4	4	3	1	1	0
Spiritists	2	1	1	0	1	1	0

Total	1998	1752	246	138	109	59	50

**Table 2:** Hypothesis 1: Private Prayer is associated with better health outcomes.

Study	Sample	Cohort followup	Predictor	Outcome	Results	Newcastle-Otawa grade
Ai et al. [25]	177 patients undergoing CABG	±3 weeks	Prayer frequencies	Postoperative complications and hospitalization	Prayer frequencies were associated with reduced complications but not hospitalization	8

Ai et al. [25]	294 patients following open-heart surgery	± 4-5 weeks	Private prayer	Quality of life	Indirect influence of using prayer for coping on quality of life through the mediation of cognitive coping and perceived social support	8

Ai et al. [27]	310 patients following open-heart surgery	± 4-5 weeks	Prayer coping	Preoperative stress symptoms	Prayer coping was inversely related to preoperative stress symptoms	7

Braam et al. [30]	2 assessment cycles: *N* = 1,702 and *N* = 1,346	3 years	Prayer frequencies	Change in depressive symptoms	At three-year followup, prayer did not predict change of depressive symptoms	7

Ai et al. [26]	246 patients awaiting cardiac surgery	2 weeks	Belief in the importance of private prayer	Optimism	Private prayer predicted optimism, along with older age, better socioeconomic resources, and healthier affect	7

Ai et al. [28]	151 older patients following CABG	1 year	Pray about their postoperative problems and private prayer	Depression and general distress	Most patients pray about their postoperative problems and that private prayer appears to significantly decrease depression and general distress one year post-CABG	8

CABG: coronary artery bypass graft surgery.

**Table 3:** Hypothesis 2: laying on of hands is associated with better health outcomes.

Study	Sample	Follow-up	Type of Study	Predictor	Outcome	Results	Jadad Score
Woods et al. [37]	65 nursing home residents	3 days	RCT, B	TT Vs PLTT	Behavioral symptoms and cortisol	Restlessness was significantly reduced in the experimental group compared to the control group (*P* = .03). There was a significant difference in morning cortisol variability among groups across time periods (<.0001)	5

Gomes et al. [47]	42 university students	3 sessions	RCT, B	TT Vs PLTT	Anxiety	The analysis of the data showed a statistically significant reduction of the state of anxiety in both groups, with *P* < .05	4

Aghabati et al. [40]	90 cancer patients undergoing chemotherapy	5 days	RCT, B	TT Vs PLTT Vs UC	Pain and Fatigue	The TT was more effective in decreasing pain and fatigue of the cancer patients undergoing chemotherapy than the usual care group, while the placebo group indicated a decreasing trend in pain and fatigue scores compared with the usual care group	5

Hawranik et al. [38]	51 residents of a long term care facility	5 days	RCT, B	TT Vs PLTT Vs UC	Disruptive behavior	Physical nonaggressive behaviors decreased significantly in those residents who received therapeutic touch compared with those who received the simulated version and the usual care. No significant differences in physically aggressive and verbally agitated behaviors were observed across the three study groups	4

Frank et al. [44]	82 patients submitted to stereotactic breast biopsy	Immediately after procedure	RCT, B	TT Vs PLTT	Pain and anxiety	No significant differences between the arms were seen regarding postbiopsy pain (*P* = .95) and anxiety (*P* = .66)	5

Woods et al. [39]	57 nursing home residents	6 days	RCT, B	TT Vs PLTT Vs UC	Overall behavioral symptoms of dementia,	The TT (significant) was more effective in decreasing behavioral symptoms of dementia than usual care, while the placebo group indicated a decreasing trend in behavioral symptoms of dementia compared to usual care	4

Blankfield et al. [45]	21 participants with carpal tunnel syndrome	6 weeks	RCT, B	TT Vs PLTT	visual analog assessments of pain and relaxation	Changes in pain scores and relaxation scores did not differ between participants in the TT group and participants in the PLTT group	4

Gordon et al. [41]	25 patients with knee osteoarthritis		RCT, B	TT Vs PLTT Vs UC	Pain and functional status	The treatment group had significantly decreased pain and improved function as compared with the placebo and control groups	4

Turner et al. [42]	99 burn patients	6 days	RCT, B	TT Vs PLTT	Pain, Anxiety and satisfaction with life	Subjects who received TT reported significantly greater reduction in pain and greater reduction in anxiety on the Visual Analogue Scale for Anxiety than did those who received sham TT	4

Meehan [46]	108 postoperative patients	1 hour	RCT, B	TT Vs PLTT Vs UC	Postoperative Pain	Therapeutic touch did not significantly decrease postoperative pain compared to the placebo control intervention	4

Keller and Bzdek [43]	60 patients with TTH	4 hours	RCT, B	TT Vs PLTT	Pain	90% of the subjects exposed to TT experienced a sustained reduction in headache pain, *P* < .0001	4

TT: therapeutic touch; PLTT: placebo therapeutic touch; UC: usual care; B: blind; RCT: randomized controlled trial; TTH: tension-type headache.

**Table 4:** Hypothesis 4: Volunteering is associated with better health outcomes.

Study	Sample	Cohort followup	Predictor	Outcome	Results	Newcastle-Otawa grade
Pillemer et al. [72]	2630 noninstitutionalized adult population	20 years	Volunteering (environmental versus other)	Physical activity, self-reported health, and depressive symptoms	Midlife environmental volunteering was significantly associated with physical activity, self-reported health, and less depressive symptoms	7

Kim and Pai [78]	2562 adults 25 years or older	8 years	Volunteering (yes/no) and frequency of volunteering	Depression	Overall, volunteering did not predict trajectories of depression; however, it affects the decline of depression for individuals above age 65	7

Krause [73]	681 older adults	6 years	Volunteering and providing tangible goods and services	Self-reported health	provision of tangible goods and services (food, clothing, shelter) to people in need were associated with better health but only for study participants who were more deeply committed to their faith	7

Ayalon [71]	5055 Israelis aged 60 years and older	7 years	Volunteering	Mortality	Those who volunteered for 10 to 14 years had a reduced mortality risk relative to nonvolunteers. In addition, those who volunteered privately, not as part of an official organization, also had a reduced mortality risk compared to nonvolunteers	9

Piliavin and Siegl [74]	4000 women and men who graduated from Wisconsin high schools	47 years	Volunteering	Psychological well-being and self-reported health	Both consistency of volunteering over time and diversity of participation are significantly related to well-being and self-reported health	8

Harris and Thoresen [69]	7527 American community-dwelling older people	96 months	Volunteering	Mortality	Frequent volunteers had significantly reduced mortality compared to nonvolunteers	8

Morrow-Howell et al. [75]	3617 adults	8 years	Volunteering	Self-rated health, functional dependency, and depression	Older adults who volunteer and who engage in more hours of volunteering report higher levels of well-being (including less depression, less functional disability, and better health)	8

Musick and Wilson [77]	2348 non-institutionalized persons aged 25 and older	5 years	Volunteering work	Depression	No association between volunteering and depression in the younger subsample; only for the 65+ age group did volunteering have a negative effect on depression, while prolonged exposure to volunteering benefits both populations	8

Van Willigen [76]	2867 adults aged 25 years or older	3 years	Volunteering	Perceived health and life satisfaction	The volunteer role is positively associated with life satisfaction and with perceived health	8

Musick et al. [70]	2348 noninstitutionalized Persons aged 25 and older	8 years	Volunteering	Mortality	Volunteering has a protective effect on mortality among those who volunteered for one organization or for forty hours or less over the past year	8

**Table 5:** Hypothesis 5: Virtuous life and Positive affect is associated with better health outcomes.

Study	Sample	Cohort followup	Predictor	Outcome	Results	Newcastle-Otawa grade	Jadad score
Wattanakit et al. [95]	12,965 middle-aged adults	9.7 years	Anger proneness	Peripheral arterial disease (PAD)	Positive association between anger proneness and incident PAD	8	—

Williams et al. [79]	12 986 adults	53 months	trait anger	Coronary heart disease (CHD)	High trait anger, compared with their low anger counterparts, were at increased risk of CHD	8	—

Xu and Roberts [83]	6856 adults	28 years	Positive Feelings	Mortality	Subjective well-being, positive feelings, life satisfaction significantly predicted lowered risks of all-cause and natural-cause mortality	8	—

Danner et al. [86]	180 Catholic nuns	53 to 73 years	Positive emotional content in early-life autobiographies	Mortality	Positive emotional content in early-life autobiographies was strongly associated with longevity 6 decades later	7	—

Davidson et al. [87]	1739 adults	10 years	positive affect	Cardiovascular events	Increased positive affect was protective against 10-year incident CHD	8	—

Versteeg et al. [93]	562 PCI patients	1 year	negative and positive affect	Health status after PCI	Baseline negative and positive affect contributes independently to patient-reported health status 12 months post-PCI	7	—

Park-Lee et al. [89]	337 caregiver and 617 noncaregiver	2 years	High and low positive affect	Frailty	Frailty risk was lower in respondents with high positive affect than in those with low positive affect in the total sample	7	—

Moskowitz et al. [84]	715 diabetes patients and 2673 controls		Positive affect	Mortality	Positive affect was significantly associated with lower risk of all-cause mortality in people with diabetes, but not in control group	7	—

Denollet et al. [88]	874 PCI patients	2 years	Positive affect	Death or myocardial infarction post-PCI	Reduced positive affect independently predicted death/MI following stent implantation	8	—

Fredman et al. [92]	432 elderly hospitalized for hip fracture	2 years	High and low positive affect	Performance-based functioning after hip fracture	High positive affect seems to have a beneficial influence on performance-based functioning after hip fracture	8	—

Ostir et al. [90]	1558 initially nonfrail older	7 years	Positive affect	Onset of frailty	positive affect is protective against the functional and physical decline associated with frailty	8	—

Fisher et al. [91]	1084 noninstitutionalized elderly with arthritis	2 years	positive and negative affect	Functional ability	High positive affect was associated with lower incidence of ADL disability in older Mexican Americans with self-reported physician-diagnosed arthritis	7	—

Moskowitz [85]	407 men who were HIV+	2 years	Positive affect	AIDS mortality	Positive affect was significantly associated with lower risk of AIDS mortality	7	—

Skodol et al. [94]	520 patients with personality disorders	4 years	Positive childhood experiences	Remission from personality disorders	Positive childhood experiences were significantly associated with remission from avoidant and schizotypal personality disorders	7	—

Yi et al. [96]	111 patients with diabetes	1 year	Resilience	HbA(1c) and self-care behaviours	Those with low or moderate resilience levels showed a strong association between rising distress and worsening HbA (1c) across time	7	—

Walter-Ginzburg et al. [97]	960 Israeli Jews aged 75–94	± 3 years	Resilience	Mortality	Resilience predicted less mortality	8	—

Poulin and Cohen Silver [104]	2138 adults	2 years	Benevolence (Goodness of the World)	Well-being	Benevolence beliefs were positively associated with well-being and that these associations were stronger with increasing age	7	—

Buchanan and Bardi [100]	86 participants	10 days	Kind acts	Satisfaction with life	Kind acts, performed daily over as little as 10 days, increased life satisfaction	7	—

Otake et al. [101]	119 participants	1 week	Counting Kindness	Happiness	People counting kindnesses increased people's subjective happiness	7	—

Carson et al. [103]	43 patients with chronic low back pain	8 weeks	Loving-kindness meditation	Pain, anger and psychological distress	Loving-kindness program reduced pain, anger, and psychological distress in patients with persistent low back pain	—	4

Hutcherson et al. [102]	93 participants	15 minutes	Loving-kindness meditation	Social connectedness	Loving-kindness meditation increased feelings of social connection and positivity toward novel individuals		4

PCI: percutaneous coronary intervention.
